# 2,2′-Dichloro-1,1′-[(propane-1,3-diyldi­oxy)bis­(nitrilo­methyl­idyne)]dibenzene

**DOI:** 10.1107/S1600536808021739

**Published:** 2008-07-19

**Authors:** Xue-Ni He, Wen-Kui Dong, Wen-Juan Bai, Hai-Bo Yan, Zhong-Wu Lv

**Affiliations:** a School of Chemical and Biological Engineering, Lanzhou Jiaotong University, Lanzhou 730070, People’s Republic of China

## Abstract

The title compound, C_17_H_16_Cl_2_N_2_O_2_, assumes a V-shape configuration with a dihedral angle between the two halves of the mol­ecule of 79.60 (4)°. The asymmetric unit comprises one half-mol­ecule with a crystallographic twofold rotation axis passing through the central C atom. There are weak inter­molecular π–π stacking inter­actions between neighbouring benzene rings with inter­molecular plane-to-plane distances of 3.277 (6) and 3.465 (5) Å along the *a* and *c* axes, respectively. In the crystal structure, weak inter­molecular C—H⋯O bonds link each mol­ecule to four others to form an infinite three-dimensional network.

## Related literature

For related literature, see: Campbell *et al.* (2001 ▶); Dong *et al.* (2006 ▶); Dong, Ding *et al.* (2008 ▶); Dong, He *et al.* (2008 ▶); Duan *et al.* (2007 ▶); Mohand *et al.* (1995 ▶); Morris *et al.* (2001 ▶); Shi *et al.* (2007 ▶).
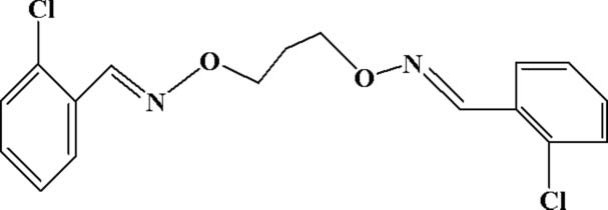


## Experimental

### 

#### Crystal data

C_17_H_16_Cl_2_N_2_O_2_
*M* *_r_* = 351.22
Orthorhombic, 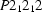

*a* = 6.5218 (7) Å
*b* = 28.586 (3) Å
*c* = 4.5120 (6) Å
*V* = 841.17 (17) Å^3^
*Z* = 2
Mo *K*α radiation
μ = 0.40 mm^−1^
*T* = 298 (2) K
0.45 × 0.18 × 0.15 mm

#### Data collection

Siemens SMART 1000 CCD area-detector diffractometer
Absorption correction: multi-scan (*SADABS*; Sheldrick, 1996 ▶) *T*_min_ = 0.842, *T*_max_ = 0.943
3761 measured reflections
1495 independent reflections
1111 reflections with *I* > 2σ(*I*)
*R*_int_ = 0.047

#### Refinement

*R*[*F* ^2^ > 2σ(*F* ^2^)] = 0.042
*wR*(*F* ^2^) = 0.086
*S* = 1.04
1495 reflections
105 parameters
H-atom parameters constrained
Δρ_max_ = 0.17 e Å^−3^
Δρ_min_ = −0.17 e Å^−3^
Absolute structure: Flack (1983 ▶), 565 Friedel pairs
Flack parameter: −0.02 (11)

### 

Data collection: *SMART* (Siemens, 1996 ▶); cell refinement: *SAINT* (Siemens, 1996 ▶); data reduction: *SAINT*; program(s) used to solve structure: *SHELXS97* (Sheldrick, 2008 ▶); program(s) used to refine structure: *SHELXL97* (Sheldrick, 2008 ▶); molecular graphics: *SHELXTL* (Sheldrick, 2008 ▶); software used to prepare material for publication: *SHELXTL*.

## Supplementary Material

Crystal structure: contains datablocks global, I. DOI: 10.1107/S1600536808021739/fl2205sup1.cif

Structure factors: contains datablocks I. DOI: 10.1107/S1600536808021739/fl2205Isup2.hkl

Additional supplementary materials:  crystallographic information; 3D view; checkCIF report

## Figures and Tables

**Table 1:** Hydrogen-bond geometry (Å, °)

*D*—H⋯*A*	*D*—H	H⋯*A*	*D*⋯*A*	*D*—H⋯*A*
C8—H8⋯O1^i^	0.93	2.55	3.479 (3)	173

Symmetry code: (i) 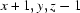.

**Fig. 1.**
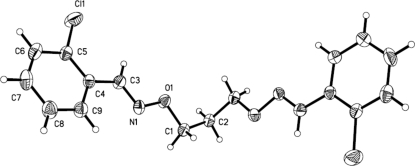
The molecular structure of the title compound with atom numbering scheme [Symmetry codes: -x + 1, -y, z]. Displacement ellipsoids for non-hydrogen atoms are drawn at the 30% probability level.

**Fig. 2.**
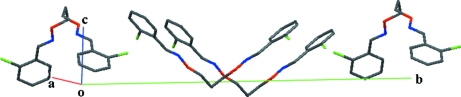
The V shape configuration of the title compound.

## supplementary crystallographic information

### Comment

Particular attention has been paid to the synthesis and study of Schiff base compounds. This is due to a variety of reasons, not the least of which is their wide application in the fields of biochemistry, synthesis and catalysis (Mohand *et al.*, 1995; Campbell *et al.*, 2001), e. g., they can easily form stable complexes with transition metal ions (Morris *et al.*, 2001). Although a great number of Schiff base compounds and their complexes have been studied crystallographically, there are only a very limited number of reports about Schiff base bisoxime compounds (Dong, Ding *et al.*, 2008; Shi *et al.*, 2007). Here we report the synthesis and crystal structure of (I) which is a bisoixime type compound.

The molecule (Fig. 1) assumes a V shape with a dihedral angle of 79.60 (4) ° between the two halves of the molecule . There is 1/2 molecule per asymmetric unit with a crystallographic twofold rotation axis passing through the central carbon (symmetry code: -*x*, -*y*, *z*) of the C1—C2—C1' unit. This structure is similar to that observed in our previously reported salen-type bisoxime compounds  (Duan *et al.*, 2007, Dong, He *et al.*2008). There are weak intermolecular π-π stacking interactions between neighbouring benzene rings with intermolecular plane-to-plane distances of  3.277 (6) and 3.465 (5) Å along the a and *c* axes, respectively (Fig. 2). In the crystal structure, weak intermolecular C—H···O hydrogen bonds (Table 1) link each molecule to 4 others to form an infinite three-dimensional network, which is different from the crystal structure of 3,3'-dibromo-1,1'-[propane-1,3-diyldioxybis(nitrilomethylidyne)]dibenzene, in which the molecules exhibit a zigzag chain array along the *a* axis formed by weak intermolecular C—H···C hydrogen bonds (Dong, Ding *et al.*, 2008).

### Experimental

The title compound was synthesized according to an analogous method reported earlier (Shi *et al.*, 2007; Dong *et al.*, 2006; Dong, Ding *et al.*, 2008). To an ethanol solution (4 ml) of 2-chlorobenzaldehyde (421.2 mg, 3.00 mmol) was added an ethanol solution (4 ml) of 1,3-bis(aminooxy)propane (155.8 mg, 1.49 mmol). The reaction mixture was stirred at 328 K for 4 h  after which the resulting precipitate was separated by filtration, and washed successively with ethanol and ethanol-hexane (1:4). The product was dried under vacuum to yield 284.7 mg of the title compound. Yield, 55.0%. mp. 361–362 K. Anal. Calc. for C_17_H_16_Cl_2_N_2_O_2_: C, 58.13; H, 4.59; N, 7.98. Found: C, 58.19; H, 4.67; N, 7.82.

Colorless needle-like single crystals suitable for X-ray diffraction  were obtained after several weeks by slow evaporation from a ethanol-chloroform solution.

### Refinement

Non-H atoms were refined anisotropically. H atoms were treated as riding atoms with distances C—H = 0.97 (CH_2_), 0.93 Å (CH), and *U*_iso_(H) = 1.2 *U*_eq_(C) and 1.5 *U*_eq_(O).

### Crystal data

**Table:**

C_17_H_16_Cl_2_N_2_O_2_	*F*_000_ = 364
*M**_r_* = 351.22	*D*_x_ = 1.387 Mg m^−^^3^
Orthorhombic, *P*2_1_2_1_2	Mo *K*α radiation λ = 0.71073 Å
Hall symbol: P 2 2ab	Cell parameters from 1292 reflections
*a* = 6.5218 (7) Å	θ = 2.9–22.2º
*b* = 28.586 (3) Å	µ = 0.40 mm^−^^1^
*c* = 4.5120 (6) Å	*T* = 298 (2) K
*V* = 841.17 (17) Å^3^	Needle-like, colorless
*Z* = 2	0.45 × 0.18 × 0.15 mm

### Data collection

**Table:**

Siemens SMART 1000 CCD area-detector diffractometer	1495 independent reflections
Radiation source: fine-focus sealed tube	1111 reflections with *I* > 2σ(*I*)
Monochromator: graphite	*R*_int_ = 0.047
*T* = 298(2) K	θ_max_ = 25.0º
φ and ω scans	θ_min_ = 1.4º
Absorption correction: multi-scan(SADABS; Sheldrick, 1996)	*h* = −7→7
*T*_min_ = 0.842, *T*_max_ = 0.943	*k* = −17→34
3761 measured reflections	*l* = −5→5

### Refinement

**Table:**

Refinement on *F*^2^	Hydrogen site location: inferred from neighbouring sites
Least-squares matrix: full	H-atom parameters constrained
*R*[*F*^2^ > 2σ(*F*^2^)] = 0.042	*w* = 1/[σ^2^(*F*_o_^2^) + (0.0338*P*)^2^] where *P* = (*F*_o_^2^ + 2*F*_c_^2^)/3
*wR*(*F*^2^) = 0.086	(Δ/σ)_max_ < 0.001
*S* = 1.05	Δρ_max_ = 0.17 e Å^−^^3^
1495 reflections	Δρ_min_ = −0.17 e Å^−^^3^
105 parameters	Extinction correction: none
Primary atom site location: structure-invariant direct methods	Absolute structure: Flack (1983), 565 Friedel pairs
Secondary atom site location: difference Fourier map	Flack parameter: −0.02 (11)

### Special details

**Table:**

Geometry. All e.s.d.'s (except the e.s.d. in the dihedral angle between two l.s. planes) are estimated using the full covariance matrix. The cell e.s.d.'s are taken into account individually in the estimation of e.s.d.'s in distances, angles and torsion angles; correlations between e.s.d.'s in cell parameters are only used when they are defined by crystal symmetry. An approximate (isotropic) treatment of cell e.s.d.'s is used for estimating e.s.d.'s involving l.s. planes.
Refinement. Refinement of *F*^2^ against ALL reflections. The weighted *R*-factor *wR* and goodness of fit *S* are based on *F*^2^, conventional *R*-factors *R* are based on *F*, with *F* set to zero for negative *F*^2^. The threshold expression of *F*^2^ > σ(*F*^2^) is used only for calculating *R*-factors(gt) *etc*. and is not relevant to the choice of reflections for refinement. *R*-factors based on *F*^2^ are statistically about twice as large as those based on *F*, and *R*- factors based on ALL data will be even larger.

### Fractional atomic coordinates and isotropic or equivalent isotropic displacement parameters (Å^2^)

**Table:**

	*x*	*y*	*z*	*U*_iso_*/*U*_eq_	Occ. (<1)
Cl1	0.68069 (12)	0.20827 (3)	0.4175 (2)	0.0826 (3)	
N1	0.8042 (3)	0.06911 (7)	0.7415 (5)	0.0441 (5)	
O1	0.6419 (2)	0.05622 (5)	0.9278 (5)	0.0502 (5)	
C1	0.6858 (3)	0.01262 (8)	1.0698 (6)	0.0454 (6)	
H1A	0.8046	0.0157	1.1976	0.054*	
H1B	0.7137	−0.0114	0.9232	0.054*	
C2	0.5000	0.0000	1.2484 (8)	0.0435 (10)	
H2A	0.5342	−0.0262	1.3754	0.052*	0.50
H2B	0.4658	0.0262	1.3754	0.052*	0.50
C3	0.7749 (4)	0.10932 (8)	0.6332 (6)	0.0489 (8)	
H3	0.6573	0.1258	0.6850	0.059*	
C4	0.9206 (3)	0.13043 (8)	0.4297 (7)	0.0414 (6)	
C5	0.8922 (4)	0.17493 (9)	0.3130 (7)	0.0467 (8)	
C6	1.0293 (5)	0.19465 (9)	0.1179 (7)	0.0583 (8)	
H6	1.0059	0.2245	0.0427	0.070*	
C7	1.2002 (5)	0.17021 (10)	0.0352 (8)	0.0674 (9)	
H7	1.2933	0.1832	−0.0975	0.081*	
C8	1.2334 (4)	0.12619 (10)	0.1497 (7)	0.0650 (9)	
H8	1.3500	0.1096	0.0950	0.078*	
C9	1.0964 (4)	0.10670 (9)	0.3433 (7)	0.0542 (8)	
H9	1.1216	0.0770	0.4185	0.065*	

### Atomic displacement parameters (Å^2^)

**Table:**

	*U*^11^	*U*^22^	*U*^33^	*U*^12^	*U*^13^	*U*^23^
Cl1	0.0880 (6)	0.0630 (5)	0.0967 (7)	0.0250 (4)	0.0168 (6)	0.0231 (5)
N1	0.0454 (12)	0.0429 (12)	0.0439 (13)	−0.0079 (11)	0.0078 (13)	−0.0029 (12)
O1	0.0508 (10)	0.0440 (9)	0.0559 (12)	−0.0012 (8)	0.0120 (11)	0.0089 (10)
C1	0.0526 (14)	0.0393 (13)	0.0443 (16)	−0.0049 (13)	−0.0008 (17)	0.0024 (14)
C2	0.053 (2)	0.039 (2)	0.038 (2)	−0.0065 (17)	0.000	0.000
C3	0.0517 (15)	0.0422 (14)	0.053 (2)	0.0016 (12)	0.0094 (16)	0.0028 (15)
C4	0.0450 (13)	0.0407 (13)	0.0384 (16)	−0.0069 (11)	−0.0002 (14)	−0.0028 (14)
C5	0.0566 (16)	0.0401 (13)	0.043 (2)	−0.0058 (13)	−0.0002 (14)	−0.0009 (14)
C6	0.079 (2)	0.0477 (16)	0.048 (2)	−0.0134 (16)	−0.0004 (18)	0.0070 (17)
C7	0.0670 (19)	0.073 (2)	0.063 (2)	−0.0248 (17)	0.019 (2)	0.0038 (19)
C8	0.0588 (18)	0.0610 (17)	0.075 (3)	−0.0040 (15)	0.0171 (18)	−0.0052 (19)
C9	0.0550 (15)	0.0454 (14)	0.062 (2)	−0.0001 (13)	0.0108 (16)	−0.0042 (16)

### Geometric parameters (Å, °)

**Table:**

Cl1—C5	1.742 (3)	C3—H3	0.9300
N1—C3	1.264 (3)	C4—C9	1.388 (3)
N1—O1	1.401 (2)	C4—C5	1.389 (3)
O1—C1	1.430 (3)	C5—C6	1.376 (4)
C1—C2	1.499 (3)	C6—C7	1.367 (4)
C1—H1A	0.9700	C6—H6	0.9300
C1—H1B	0.9700	C7—C8	1.377 (4)
C2—C1^i^	1.499 (3)	C7—H7	0.9300
C2—H2A	0.9700	C8—C9	1.368 (4)
C2—H2B	0.9700	C8—H8	0.9300
C3—C4	1.453 (3)	C9—H9	0.9300
			
C3—N1—O1	110.9 (2)	C9—C4—C3	121.0 (2)
N1—O1—C1	110.30 (16)	C5—C4—C3	122.2 (2)
O1—C1—C2	106.78 (17)	C6—C5—C4	122.1 (3)
O1—C1—H1A	110.4	C6—C5—Cl1	117.6 (2)
C2—C1—H1A	110.4	C4—C5—Cl1	120.3 (2)
O1—C1—H1B	110.4	C7—C6—C5	119.7 (3)
C2—C1—H1B	110.4	C7—C6—H6	120.2
H1A—C1—H1B	108.6	C5—C6—H6	120.2
C1^i^—C2—C1	115.0 (3)	C6—C7—C8	119.5 (3)
C1^i^—C2—H2A	108.5	C6—C7—H7	120.3
C1—C2—H2A	108.5	C8—C7—H7	120.3
C1^i^—C2—H2B	108.5	C9—C8—C7	120.6 (3)
C1—C2—H2B	108.5	C9—C8—H8	119.7
H2A—C2—H2B	107.5	C7—C8—H8	119.7
N1—C3—C4	121.6 (2)	C8—C9—C4	121.3 (3)
N1—C3—H3	119.2	C8—C9—H9	119.3
C4—C3—H3	119.2	C4—C9—H9	119.3
C9—C4—C5	116.8 (3)		
			
C3—N1—O1—C1	−174.7 (2)	C3—C4—C5—Cl1	2.0 (4)
N1—O1—C1—C2	−176.4 (2)	C4—C5—C6—C7	−0.3 (4)
O1—C1—C2—C1^i^	68.74 (16)	Cl1—C5—C6—C7	178.4 (2)
O1—N1—C3—C4	−179.3 (2)	C5—C6—C7—C8	−0.4 (5)
N1—C3—C4—C9	1.0 (4)	C6—C7—C8—C9	0.5 (5)
N1—C3—C4—C5	−178.8 (3)	C7—C8—C9—C4	0.1 (4)
C9—C4—C5—C6	0.8 (4)	C5—C4—C9—C8	−0.7 (4)
C3—C4—C5—C6	−179.3 (2)	C3—C4—C9—C8	179.4 (2)
C9—C4—C5—Cl1	−177.9 (2)		

Symmetry codes: (i) −*x*+1, −*y*, *z*.

### Hydrogen-bond geometry (Å, °)

**Table:**

*D*—H···*A*	*D*—H	H···*A*	*D*···*A*	*D*—H···*A*
C8—H8···O1^ii^	0.93	2.55	3.479 (3)	173

Symmetry codes: (ii) *x*+1, *y*, *z*−1.

## References

[bb1] Campbell, E. J., Zhou, H. & Nguyen, S. T. (2001). *Org. Lett. ***3**, 2391–2393. 10.1021/ol0162116 11463324

[bb2] Dong, W.-K., Ding, Y.-J., Luo, Y.-L., Lv, Z.-W. & Wang, L. (2008). *Acta Cryst.* E**64**, o1324. 10.1107/S1600536808018187 PMC2961867 21202948

[bb3] Dong, W. K., Feng, J. H. & Yang, X. Q. (2006). *Z. Kristallogr. New Cryst. Struct.* **221**, 447–448.

[bb4] Dong, W.-K., He, X.-N., Zhong, J.-K., Chen, X. & Yu, T.-Z. (2008). *Acta Cryst.* E**64**, o1098. 10.1107/S1600536808012701 PMC2961531 21202612

[bb5] Duan, J.-G., Dong, C.-M., Shi, J.-Y., Wu, L. & Dong, W.-K. (2007). *Acta Cryst.* E**63**, o2704–o2705.

[bb6] Flack, H. D. (1983). *Acta Cryst.* A**39**, 876–881.

[bb7] Mohand, S. A., Levina, A. & Muzart, J. (1995). *J. Chem. Res. (S)*, **25**, 2051–2058.

[bb8] Morris, G. A., Zhou, H., Stern, C. L. & Nguyen, S. T. (2001). *Inorg. Chem.* **40**, 3222–3227. 10.1021/ic010090o 11399196

[bb9] Sheldrick, G. M. (1996). *SADABS* University of Göttingen, Germany.

[bb10] Sheldrick, G. M. (2008). *Acta Cryst.* A**64**, 112–122. 10.1107/S0108767307043930 18156677

[bb11] Shi, J., Dong, W., Zhang, Y. & Gao, S. (2007). *Acta Cryst.* E**63**, o4080.

[bb12] Siemens (1996). *SMART* and *SAINT* Siemens Analytical X-ray Instruments Inc., Madison, Wisconsin, USA.

